# Analysis of Genes Related to Invadopodia Formation and CTTN in Oral Squamous Cell Carcinoma—A Systematic Gene Expression Analysis

**DOI:** 10.3390/cimb45080437

**Published:** 2023-08-18

**Authors:** Immanuel Desel, Susanne Jung, Nikolai Purcz, Yahya Açil, Christoph Sproll, Johannes Kleinheinz, Sonja Sielker

**Affiliations:** 1 Vascular Biology of Oral Structures (VABOS) Research Unit, Department of Cranio-Maxillofacial Surgery, University Hospital Muenster, 48149 Muenster, Germany; i_dese01@uni-muenster.de (I.D.); susanne.jung@ukmuenster.de (S.J.); johannes.kleinheinz@ukmuenster.de (J.K.); 2 Department of Cranio-Maxillofacial Surgery, University Hospital Kiel, 24105 Kiel, Germanyacil@mkg.uni-kiel.de (Y.A.); 3 Department of Cranio-Maxillofacial Surgery, University Hospital Duesseldorf, 40225 Duesseldorf, Germany

**Keywords:** OSCC, *CTTN*, invadopodia, oral squamous cell carcinoma, cortactin, gene expression, invasion, metastasis

## Abstract

Successful treatment for any type of carcinoma largely depends on understanding the patterns of invasion and migration. For oral squamous cell carcinoma (OSCC), these processes are not entirely understood as of now. Invadopodia and podosomes, called invadosomes, play an important role in cancer cell invasion and migration. Previous research has established that cortactin (*CTTN*) is a major inducer of invadosome formation. However, less is known about the expression patterns of *CTTN* and other genes related to it or invadopodia formation in OSCC during tumor progression in particular. In this study, gene expression patterns of CTTN and various genes (n = 36) associated with invadopodia formation were analyzed to reveal relevant expression patterns and give a comprehensive overview of them. The genes were analyzed from a whole genome dataset of 83 OSCC samples relating to tumor size, grading, lymph node status, and UICC (Union for Internatioanl Cancer Control). The data revealed significant overexpression of 18 genes, most notably *CTTN*, *SRC* (SRC proto-onocogene, non-receptor tyrosine kinase)*, EGFR* (epidermal growth factor receptor), *SYK* (spleen associated tyrosine kinase), *WASL* (WASP like actin nucleation promotion factor), and *ARPC2* (arrestin beta 1) due to their significant correlation with further tumor parameters. This study is one of the first to summarize the expression patterns of *CTTN* and related genes in a complex group of OSCC samples.

## 1. Introduction

Oral squamous cell carcinoma (OSCC) is a highly prevalent cancer, coinciding with high morbidity and mortality rates. It was diagnosed over 375,000 times and caused more than 175,000 deaths internationally in 2020 alone [1]. In spite of good accessibility for visual inspection and biopsy, oral cancer is detected at advanced stages in 64% of patients. High invasiveness and metastasis of OSCC contribute negatively to disease progression; the 5-year survival rates of patients with regional and distant forms of oral cancer lie at 66% and 39% [2].

In the epithelial-to-mesenchymal transition (EMT) process, cancer cells acquire invasive and migratory properties by losing cell–cell adhesions and disintegrating the extracellular matrix (ECM). Invadopodia, lamellipodia and filopodia, so-called invadosomes, are crucial for cell motility and intravasation [3,4]. Cortactin (CTTN) has been identified as one inducer for EMT by being part of and interacting with signaling pathways relevant to the formation of invadopodia [5]. These invadopodia consist of an F-actin core coupled with actin regulators such as CTTN, Arp2/3 complex and neuronal Wiskott–Aldrich syndrome protein (WASL); they further rely on matrix metalloproteinases (MMPs), mainly MMP14, to degrade the ECM [6,7]. Additional participants are upstream regulators like WASL interacting protein (WIP), SRC, CDC42, EGF and EGFR, MET and the tyrosine kinase substrate scaffolding proteins TKS4 and TKS5 consisting of four and five Src-homology-3 domains, respectively [8,9].

Previous studies have explored genes associated with invasion and migration in OSCC, and increasingly, they are becoming therapeutic targets and prognostic markers [10]. However, there is a gap in current OSCC research regarding a comprehensive analysis of multiple genes relevant for cancer cell migration and invasion from a single sample pool. The aim of our study was to fill this gap by investigating the expression patterns of a broader spectrum of genes.

Our focus lays on genes related to OSCC invasion and spread, as it is owing to these characteristics that OSCC belongs to the deadlier human cancers. For this, we summarized gene expression patterns of *CTTN* and genes related to it, as well as other genes responsible for invasion mechanics of OSCC cancer cells.

We sought to provide detailed information on expression patterns and their correlation with various tumor parameters including size, grading, lymph node association and UICC classification (Union for International Cancer Control).

By doing so, we aimed to enhance our understanding of the interplay between these genes and the influence of tumor parameters, ultimately contributing to a more comprehensive understanding of OSCC progression.

This overview of gene expression patterns is based on whole-genome-microarray data and no single expression via real-time qPCR. We plan to build up from these data; nonetheless, we also want to share these results with the scientific community as an opportunity to compare their results and further elaborate on the provided data.

## 2. Materials and Methods

### 2.1. Patient Data

For this retrospective analysis, 83 tissue samples were taken during tumor surgery after informed consent of the patients in the years 2009–2012. Inclusion criteria were patients over the age of 18, with a histologically diagnosed oral squamous cell carcinoma, and no adjuvant radiation or chemotherapy. Patients with recurrent disease were included. Healthy tissue controls (n = 30) were taken from oral vestibular mucosa samples during orthognathic or traumatology surgery after informed consent. The tissue samples were snap-frozen in liquid nitrogen after surgery and stored at −80 °C until further usage [11,12].

The Ethics Committee of the medical faculty approved the study setup; the ID of the ethical clearance (WWU Muenster) is 2008-580-f-s, and the study is registered in a public Clinical Trials Registry, DRKS00000199.

### 2.2. RNA Extraction and Microarray Assay

The whole study design, including RNA extraction, microarray assay, and bioinformation steps were described before [11,12]. In brief, total RNA was prepared with the miRNeasy Mini Kit (Qiagen, 40724 Hilden, Germany). For microarray analysis, we used the Agilent Array platform employing the manufacturer’s standard protocols for sample preparation and microarray hybridization. Gene expression analysis was performed with the Whole Human Gene Expression Microarray (4 × 44K; GPL4133), arrays were scanted with the Agilent G2505B Microarray Scanner and feature extraction was performed with Feature Extraction software version 9.5 (all Agilent Technologies, 76337 Waldbronn, Germany). Data files from mRNA microarrays were analyzed by GeneSpring GX 7.3.1 according to the manufacturer’s protocol (Agilent Technologies, 76337 Waldbronn, Germany). The first normalization step consisted of background elimination while, in a second step, the 50th percentile of each spot was normalized. Normalizations to a healthy oral mucosa pool was performed in the last step with the expression factor for the healthy oral mucosa pool set to 1; the fold change to control is presented in the tables. Primary statistical analysis was performed with GeneSpring GX 7.3.1 software (Agilent Technologies, 76337 Waldbronn, Germany).

### 2.3. Identification of Invadopodia-Related Genes

In this study, we wanted to focus on genes related to OSCC invasion and spread, as it is owing to these characteristics that OSCC belongs to the deadlier human cancers. At first, cortactin was singled out by reading multiple studies found in the PubMed database under the search terms “oscc AND invasion”. The list of related and relevant genes was assembled through two methods. Firstly, we read all available research found under the terms “oscc AND cortactin”, “oscc AND cttn”, “hnscc AND cortactin” and “hnscc AND cttn” to gather further relevant genes. Secondly, we screened all the interactions of cortactin in the NCBI Gene database and searched for these genes in studies on PubMed. For the search algorithm, each gene was coupled with each of these four different keywords: OSCC, HNSCC, Cortactin and Invadopodia. The final genes were selected based on the amount of relevant hits in these categories. A total of 37 genes were selected

### 2.4. Statistical Analysis

The statistical analysis was performed using the statistical software SPSS version 28 (IBM, 71139 Ehningen, Germany). Due to the non-normal and non-homogenous distribution of the data, the median test was used. The level of significance was set at *p* < 0.05. Parameters were UICC classification, T status, lymph node status, grading, smoker, alcohol abuse and type of OSCC. For the comparison of gene expression between two groups inside these parameters, the chi-square test was used. Only 11 of 37 genes displayed statistically significant expression patterns and were included in the discussion, except for *AFAP1*. 

## 3. Results

### 3.1. Overview

During 2009 and 2012, a whole genome analysis of 83 OSCC samples was performed. Detailed information about patient data and tumor sample preparation were described before [11,12]. An overview of all the included tumor parameters is presented in Table 1.

A huge dataset was generated that enabled the systematical analysis of a multitude of questions regarding the molecular science of OSCC.

For this study, a pool of 37 genes associated with initiating cancer cell migration, and thus metastasis, was analyzed regarding their overall expression as summarized in Table 2. The main objective was to take a broader spectrum of genes and give a comprehensive overview and comparison regarding their impact on disease progression and prognosis, specifically for OSCC.

A further analysis inside the subcategories from Table 1 was conducted to confirm and reveal the influence of size, grading, lymph node metastasis, lifestyle factors, gender and type of OSCC on gene expression profiles. A comprehensive overview of our results is given in Table 3 and Table 4, where the fold changes in downregulated genes (n = 11) and of upregulated genes (n = 20) are listed.

### 3.2. Gene Expression Patterns

Beginning with *CTTN*, 57 out of 83 samples (69%) showed an overexpression, surpassing the established 0.95 baseline. There was a significant (*p* = 0.02) difference in tumor size, with 61% of T1 and T2 samples showing an overexpression and 85% of T3 and T4 samples. The mean value of expression increased from 2.2 (T1 + T2) to 2.8 (T3 + T4), indicating an upregulation in later tumor stages.

Among the 83 samples, 63 (76%) demonstrated overexpression of the *SRC* gene. Notably, there was an increase in the proportion of overexpressed *SRC* with a higher tumor grade. Due to the insufficient sample size of G1 (n = 2), it was not taken into account. However, there was a substantial increase in expression from G2 at 74% to G3 at 92% of samples overexpressed. 

*EGFR* was found to be overexpressed in a total of 79 samples (95%), also exhibiting a significant (*p* = 0.045) difference in distribution between tumor sizes. A total of 93% of T1 + T2 samples were overexpressed, while every single T3 + T4 sample (100%) was overexpressed, compared to the 0.95 baseline.

*SYK* was analyzed due to its involvement in integrin signaling and the formation of *WAVE* and *WASP* complexes [13,14]. An amount of 67 samples (81%) showed overexpression with a significant (*p* = 0.049) increase from 75% of samples for T1 + T2 to 93% for T3 + T4.

Among the analyzed samples, the *WASL* gene was overexpressed in 53 (64%). Although the increase was not statistically significant (*p* = 0.054), it should still be mentioned that only 57% of T1 + T2 samples were overexpressed but 78% of T3 and T4 samples were. Thus, this also suggests a potential association with tumor progression for *WASL*.

Though *AFAP1* was only overexpressed in 60% of samples, there was a significant (*p* = 0.038) increase in grading. It increased from G2 at 54% to G3 at 85% of samples overexpressed. 

*ARPC2* was overexpressed in 69% of samples and increased significantly (*p* = 0.02) from T1 + T2 (61%) to T3 + T4 (85%). Additionally, *ARPC2* increased significantly (*p* = 0.031) from UICC 1 + 2 (57%) to UICC 3 + 4 (78%).

Several genes displayed overexpression with a 95% confidence level (*p* < 0.05). These were *PTK2*, *MET*, *STAT1*, *GRB2*, *MAPRE1*, *PLAUR*, *FLNA*, *WIPF1*, *TKS4*, *FSCN1*, *GDI1*, *CDC42*, *ACTR2*, and *ACTR3*.

Of these genes, *FSCN1* and *TKS4* were especially noteworthy with an overexpression in 98% of samples.

A smaller number of genes displayed a significant (*p* < 0.05) downregulation. The genes observed were *CTTNBP2*, *HDAC6* isoform b and c, *ARRB1*, *SIRT1*, *SHANK1*, *SHANK2*, *MMP14*, and *ACTR3B*. They all exhibited reduced expression levels compared to healthy oral mucosa.

Genes differing insignificantly from healthy tissue were *IMP-3*, *CDH1*, *IQGAP1*, *NEDD9*, *TKS5*, *WAS* and *ARPC4*, indicating minimal alterations in their expression levels in the analyzed cancer samples.

### 3.3. Statistical Visualization

Additional data for the differentially expressed genes (DEG) can be found in the following Table 3 and Table 4, depicting the fold changes in all DEGs. The percentages of overexpressed samples, in combination with the mean values across the respective categories, allow for a thorough quantitative report.

We added the standard deviation to allow for a clearer perspective on the fluctuation of gene expression. The mean values are to be understood in reference to the 0.95 baseline set according to the expression levels in our pool of healthy oral mucosa samples.

## 4. Discussion

EMT and invadopodia formation play a crucial role in multiple human carcinomas and lead to metastasis and unfavorable disease progression [15,16,17]. Unsurprisingly, it has been the topic of thorough research and bears the potential to improve means of prognosis, diagnosis and treatment [18,19]. With breast and ovarian cancer as main topics of EMT research, we want to give an overview of genes relevant for invadopodia formation, with a lesser focus on OSCC, and review the current state of research. In the following part, a selection of the observed genes regarding their expression and potential for future clinical application will be discussed.

In accordance with current literature, we observed a significant overexpression of *CTTN* in our tumor samples, comparing it to the healthy mucosa samples.

The location of the *CTTN* gene on chromosome 11q13, a region frequently amplified in OSCC and associated with worse prognosis, further supports its significance for this type of cancer [20,21]. It encodes cortactin, which serves as an F-actin-binding protein (ABP) and has been found to be overexpressed in human carcinomas as early as 1992 [22].

The overexpression observed in our date became especially apparent in advanced tumor stages (T3 and T4), suggesting a correlation with disease progression.

Furthermore, the significant increase in expression with growing tumor size (pT) highlights its potential as a prognostic marker for OSCC.

A significant difference regarding the stage of lymph node metastasis (pN) could not be observed, however. The lack of a significant difference in CTTN expression regarding pN could be attributed to the timeframe between initial degradation of the ECM and the point at which a change in pN becomes clinically diagnoseable. An overexpression of invadopodia-associated genes could considerably precede a metastatic manifestation, since invadopodia are involved in the earliest stages of EMT [23,24].

Considering the prognostic and diagnostic value of CTTN expression, a recent clinical study by Boeve et al. demonstrated that it can help identify patients with occult metastasis [25].

Despite sentinel lymph node biopsy (SLNB) and neck dissection (ND) remaining the best diagnostic tools for assessing pN, cortactin expression can help decide which low-risk patients might benefit from a less invasive “watch and see” strategy. Further clinical research is needed and a combination of tumor biomarkers bares potential to raise diagnostic reliability.

Moving on to the *SRC* gene, our findings revealed an overexpression in the majority of OSCC samples. The non-receptor tyrosine kinases of the SRC family play a vital role in cancer research and c-SRC has been linked to cancer cell motility, invasiveness and an increase in regional lymph node metastasis in OSCC and HNSCC [26,27].

The increased expression of *SRC* with higher tumor grading (G3) suggests its potential involvement in tumor aggressiveness and progression. These findings indicate that *SRC* may contribute to the invasive properties of OSCC cells and their ability to metastasize. Due to its nature as an upstream regulator, in the following part, we will briefly highlight the SRC substrates involved in invadopodia formation and actin regulation.

Unphosphorylated CTTN can bind to F-actin and the Arp2/3 complex, which is responsible for the nucleation of actin branches in invadopodia [28]. Thus, cortactin induces actin nucleation and simultaneously stabilizes newly formed actin branches [29].

WIP can also bind to unphosphorylated CTTN and increase its Arp2/3 complex activation capability [30]. However, Src-mediated tyrosine phosphorylation of cortactin enables the additional binding of the adaptor protein Nck1, which leads to substantially higher Arp2/3 activation [31]. Cortactin and Nck1 can also bind to and enhance the activity of N-WASp, which is another important recruiter of the Arp2/3 complex [32,33].

Two additional c-Src substrates, the scaffold proteins Tks4 and Tks5, are involved in the formation and maturation of invadopodia [34,35]. Tks5 mediates the binding of an invadopodia precursor complex to Phosphatidylinositol 4-5-bisphosphate (PIP_2_). Arp2/3, Cofilin and N-WASp form around an actin–cortactin core to make up this precursor complex, which is only stabilized after being connected to the plasma membrane [7,36].

In absence of Tks4 and Tks5, the formation of podosomes and thus invadopodia, as well as ECM degradation, are disrupted. The latter due to MT1-MMP not being recruited to the podosomes. After reintroducing Tks4 and Tks5, respectively, the podosome formation returned to normal; however, only Tks4 led to the return of MT1-MMP and thereby ECM degradation [35].

In our dataset, *TKS4* showed a clear overexpression in 95% of samples; *TKS5* levels did not differ significantly from the healthy baseline.

However, multiple researchers showed that *TKS5* is an important gene required for invadopodia formation and is overexpressed in different types of human cancers, including OSCC [37,38].

Tks5 isoforms lacking the phox homology (PX) domain have gotten more attention and research recently. These PX-domain-lacking Tks5 isoforms were coined Tks5β and Tks5short.

Multiple studies strongly suggest the participation of Tks5long in invadopodia formation [39,40].

In lung adenocarcinoma, the ratio of Tks5long to Tks5short correlates with invadopodia formation and metastasis. Only Tks5long seems to promote invadopodia formation, while Tks5short acts as an inhibitor of this process [40].

These circumstances beg the question of why our dataset could not confirm this trend in the OSCC samples at hand. The array we used showed isoform 1 of the *TKS5* mRNA with the NM accession number NM_014631.3, which translates into the protein TKS5long.

The differentiation between TKS5long and TKS5β/TKS5short happens through alternative transcription, ruling out an observation through our array data.

*CDC42*, a member of the Rho-family GTPases, represents another regulator of N-WASp and was found to be overexpressed in 82% of our OSCC samples.

Together with *PIP_2_*, Cdc42 functions as an upstream regulator of N-WASp. In its base state, the terminal NH_2_- and -COOH groups of N-WASp obturate its ARP2/3 binding site by interacting with one another. Through binding on specific regions of N-WASp, Cdc42 and PIP_2_ inhibit this interaction and expose the ARP2/3 binding site [41].

The elevated Cdc42 expression in relation with elevated N-WASp suggests an increased activity of this pathway in OSCC and a possible target for pharmacotherapy.

Furthermore, Cdc42, together with RhoA, enables the accumulation of MMP14, also known as MT1-MMP in the invadopodium, with the purpose of degrading the ECM [42]. Though MMP14 has been found to be overexpressed in OSCC samples in various studies, our data showed an opposing expression profile with 80% of samples underexpressing *MMP14* [38,43].

In the dataset, a noticeable but statistically insignificant increase in *AFAP1* was observed. Nonetheless, further research for OSCC should consider taking a deeper look at it, primarily because of its function as an upstream regulator of *SRC*, as well as its involvement in cytoskeletal activity [44,45].

The subunit ARPC2 is a major structural component of the ARP2/3 complex and an elevated expression has been associated with metastasis, tumor size and lymph node invasion in other types of cancer [46,47]. Regarding our OSCC tissue samples, *ARPC2* showed comparable results regarding tumor size, with a 24% increase from T1+2 to T3+4 as well as a 21% increase from UICC 1 + UICC 2 to UICC 3 + UICC 4. A significant change in expression levels could not be observed for lymph node invasion or metastasis. However, transferring research approaches of Arp2/3 and its subunits from other types of cancers could still prove beneficial for OSCC research.

One such promising discovery is the off-label use of the commercially available antipsychotic drug Pimozide. Choi J, Lee YJ, Yoon YJ, et al. were able to show that Pimozide inhibited the interaction between ARPC2 and vinculin in a DLD-1 colon cancer cell line, resulting in an absence of cortactin and lamellipodia at the leading edge of the cell [48].

The significant overexpression of FAK/PTK2 points towards an increased activity in cell migration. Decreased FAK/PTK2 activity leads to a slower disassembly of cell/cell junctions during EMT, and through multiple mechanisms controls cell/ECM adhesion [49,50,51]. Especially interesting is the binding of cortactin to the C-terminus of FAK, since a mutation of this binding site leads to a decreased turnover rate of cell/ECM adhesions. Even though the two relevant proline-rich sequences also interact with other binding partners, cortactin might be the most important one [52].

Another major factor in various human cancers is the epidermal growth factor (EGF) and its correlating receptor, the epidermal growth factor receptor (EGFR).

EGFR is a transmembrane receptor protein involved in cell growth and proliferation. In normal cells, EGFR is activated when its ligand EGF binds to it, which leads to the activation of intracellular signaling pathways that promote growth, differentiation, and survival of the cell [53]. *EGFR* has been found to be overexpressed in multiple types of cancer, including OSCC. Overexpression of *EGFR* in OSCC has been associated with poor prognosis and increased invasion and metastasis. This overexpression can be caused by different processes such as gene amplification, mutation, or increased protein synthesis [54,55,56]. The mutations that have been found to occur in *EGFR* regarding OSCC are mainly in-frame deletions, missense mutations and insertions which occur in exon 19 and exon 21 of the EGFR gene [57,58]. Our results showed an overexpression of *EGFR* in 93% of T1 + T2 samples, and in 100% of the T3 + T4 samples. These results are in accordance with current literature regarding overexpression, but the amount of tissue samples showing an overexpression differs from study to study. Some of these differences could fall back on different methods of testing for overexpression, e.g., immunohistochemistry on formalin-fixated cells or fluorescent in situ hybridization, which should be considered as the gold standard concerning reproducibility and accuracy [59].

EGFR is known as a prognostic marker for OSCC, though it has a wavering prognostic value. But instead of being used as a prognostic marker, it seems to be a promising target for treating OSCC. Several EGFR inhibitors were developed and are currently undergoing clinical trials. These inhibitors include monoclonal antibodies that can inhibit the intracellular signaling of EGFR by binding to the tyrosine kinase (TK) section of EGFR. Some of these so-called tyrosine kinase inhibitors (TKIs), like afatinib, erlotinib, gefitinib, and osimertinib, are already approved for treating patients with EGFR-mutated non-small-cell lung cancer [60]. Gefitinib is one of the most promising therapeutic agents targeting EGFR in OSCC [61]. It was also recently shown to improve prognosis in chemoresistant CYLD negative OSCC patients [62].

It is important to note that despite our results, not all OSCC patients will have EGFR mutation. Such treatments will not show any effects, but there is research ongoing to understand the role of EGFR in OSCC and how to best target it. Additionally, EGFR inhibitors can have side effects and their efficacy may be limited by the development of resistance; so, treatment must be tailored to each individual patient and needs to be closely monitored.

## 5. Conclusions

Our research’s goal was to shed light on the significant role of genes responsible for invasiveness in OSCC. We have identified potential biomarkers through a comprehensive analysis of gene expressions, most notably, *CTTN*, *SRC*, *TKS4*, and *CDC42*, which showed consistent overexpression patterns and strong association with disease progression.

These findings highlight the correlation of *CTTN* expression regarding multiple tumor parameters in OSCC, especially with rising expression in advanced tumor stages, suggesting its potential as a reliable indicator of disease severity. Additionally, the overexpression of SRC and its associated substrates underscores their involvement in cancer cell motility, invasiveness, and lymph node metastasis, implicating them as crucial players in OSCC progression and spread.

*TKS4* and *CDC42* have emerged as promising biomarkers, presenting significant overexpression and indicating their potential as prognostic indicators for OSCC aggressiveness. Furthermore, our analysis identified additional genes worth mentioning, including *PTK2*, *MET*, *STAT1, GRB2*, *MAPRE1*, *PLAUR*, *FLNA*, *WIPF*, *FSCN1*, *GDI1*, *ACTR2*, and *ACTR3*. This broad range of genes may hold important diagnostic and prognostic value in OSCC.

These findings have the potential to assist in the clinical management of OSCC, enabling improved prognosis, more accurate diagnosis, and the development of targeted treatment strategies. However, further research and validation studies are crucial to explore the clinical applications of these genes and their specific roles in OSCC progression and metastasis.

Overall, we hope to contribute to a deeper understanding of the molecular mechanisms underlying OSCC and pave the way for future investigations into novel therapeutic targets and more personalized approaches for patients with this devastating disease.

## Figures and Tables

**Table 1:** Overview of observed OSCC parameters.

Group		Number	(%)
T-status	T1 + T2	56	(67)
	T3 + T4	27	(33)
G-status	G1	2	(2.4)
	G2	67	(80.7)
	G3	14	(16.9)
N-status	N−	53	(64)
	N+	30	(36)
UICC classification (TNM 7th edition; 2009)	UICC1	15	(18.1)
	UICC2	23	(27.7)
	UICC3	8	(9.6)
	UICC4	37	(44.6)
Type of OSCC	keratinized	67	(81)
	not-keratinized	9	(11)
	n/a	7	(8)
Smoker	Yes	49	(59)
	No	31	(37)
	n/a	3	(4)
Alcohol abuse	Yes	48	(58)
	No	32	(39)
	n/a	3	(4)
Smoking and alcohol		40	(48)
Localization	mouth floor	23	(27.7)
	alveolar ridge	22	(25.4)
	tongue	20	(24)
	buccal plain	8	(9.6)
	lip	5	(6)
	palate	3	(3.6)

Note: One recurrent tumor in T1 group.

**Table 2:** Selected gene expressions in all 83 OSCC samples.

Gene Symbol (Name)	RefSeq Transcript	Downregulated ^1^ (%)	Upregulated ^1^ (%)
*CTTN*	NM_005231	31	69
*CTTNBP2*	NM_03427	100	0
*SRC*	NM_005417	24	76
*EGFR*	NM_005228	5	95
*PTK2*	NM_153831	19	81
*IMP-3*	NM_006547	47	53
*MET*	NM_000245	8	92
*CDH1*	NM_004360	55	45
*STAT1*	NM_007315	6	94
*SYK*	NM_003177	19	81
*HDAC6*	NM_006044	85	15
*GRB2*	NM_002086	15	85
*MAPRE1*	NM_012325	2	98
*PLAUR*	NM_001005377	7	93
*IQGAP1*	NM_003870	39	61
*NEDD9*	NM_006403	61	39
*ARRB1*	NM_004041	93	7
*FLNA*	NM_001456	35	65
*SIRT1*	NM_012238	75	25
*SHANK1*	NM_016148	93	7
*SHANK2*	NM_012309	86	14
*WIPF1*	NM_003387	20	80
*WAS*	NM_000377	60	40
*WAS-L*	NM_003941	36	64
*TKS4*	NM_001017995	5	95
*TKS5*	NM_014631	58	42
*MMP14*	NM_004995	80	20
*FSCN1*	NM_003088	2	98
*AFAP1*	NM_021638	40	60
*ARPC2*	NM_152862	31	69
*ARPC4*	NM_005718	55	45
*GDI1*	NM_001493	11	89
*CDC42*	NM_044472	18	82
*ACTR2*	NM_001005386	13	87
*ACTR3*	NM_005721	14	86
*ACTR3B*	NM_020445	78	22

^1^ Percentage of OSCC samples under or over the reference set by healthy tissue samples.

**Table 3:** Expression patterns of downregulated genes related to invadopodia formation in at least 60% of all OSCC samples compared to healthy mucosa (± = standard deviation; % = percent in OSCC samples, T = T status, G = grading, N = lymph nodes, UICC = UICC classification; * group G1 includes two samples, results are less robust; ^#^ genes are upregulated in group G1).

Gene	All	T1 + T2	T3 + T4	G1 *	G2	G3	N0	N+	UICC1	UICC2	UICC3	UICC4
	n = 83	n = 56	n = 27	n = 2	n = 68	n = 13	n = 53	n = 30	n = 16	n = 23	n = 8	n = 36
*CTTNBP2*	0.14	0.16	0.10	0.13	0.17	0.14	0.14	0.16	0.13	0.14	0.14	0.14
NM_033427	±0.14	±0.16	±0.09	±0.09	±0.139	±0.16	±0.12	±0.18	±0.15	±0.09	±0.1	±0.17
	100%	100%	96%	100%	100%	100%	100%	100%	100%	100%	100%	100%
*HDAC6*	0.61	0.62	0.60	0.92	0.60	0.65	0.61	0.60	0.67	0.59	0.61	0.60
NM_006044	±0.31	±0.17	±0.15	±0.1	±0.16	±0.17	±0.16	±0.16	±0.17	±0.18	±0.19	±0.14
isoform b	86%	82%	93%	100%	88%	77%	83%	90%	86%	83%	100%	84%
*HDAC6*	0.63	0.67	0.56	0.72	0.61	0.71	0.62	0.64	0.65	0.69	0.61	0.59
BC011498	±0.20	±0.18	±0.21	±0.10	±0.19	±0.20	±0.19	±0.21	±0.20	±0.14	±0.18	±0.23
isoform c	78%	77%	81%	100%	78%	77%	79%	77%	86%	78%	100%	71%
*NEDD9*	0.47	0.5	0.43	1.97 ^#^	0.48	0.43	0.49	0.44	0.49	0.48	0.49	0.45
NM_006403	±0.25	±0.27	±0.23	±0.64	±0.26	±0.22	±0.25	±0.25	±0.33	±0.26	±0.22	±0.24
	61%	54%	78%	100%	66%	46%	60%	63%	57%	61%	75%	61%
*ARRB1*	0.41	0.41	0.42	0.67	0.40	0.40	0.42	0.40	0.50	0.35	0.37	0.42
NM_004041	±0.22	±0.22	±0.23	±0.3	±0.22	±0.19	±0.24	±0.19	±0.290	±0.159	±0.185	±0.227
	93%	95%	89%	100%	93%	92%	89%	100%	86%	96%	88%	95%
*SIRT1*	0.70	0.67	0.72	1.17 ^#^	0.70	0.72	0.70	0.70	0.63	0.70	0.68	0.73
NM_012238	±0.17	±0.16	±0.17	±0.68	±0.17	±0.13	±0.18	±0.14	±0.19	±0.19	±0.21	±0.14
	75%	68%	89%	100%	78%	69%	72%	80%	57%	74%	88%	79%
*SHANK1*	0.40	0.49	0.41	1.34	0.40	0.40	0.40	0.41	0.51	0.34	0.47	0.38
NM_016148	±0.24	±0.26	±0.22		±0.25	±0.20	±0.24	±0.26	±0.29	±0.21	±0.25	±0.24
	93%	95%	89%	50%	93%	100%	92%	93%	93%	91%	100%	92%
*SHANK2*	0.28	0.28	0.28	1.145	0.29	0.25	0.30	0.24	0.17	0.33	0.31	0.28
NM_012309	±0.23	±0.24	±0.22		±0.23	±0.22	±0.25	±0.18	±0.18	±0.27	±0.28	±0.20
	86%	83%	92%	50%	87%	85%	84%	89%	85%	86%	88%	86%
*WAS*	0.72	0.74	0.65	1.02	0.72	0.65	0.74	0.67	0.68	0.80	0.60	0.70
NM_000377	±0.15	±0.15	±0.16		±0.75	±0.18	±0.51	±0.15	±0.15	±0.13	±0.19	±0.16
	60%	68%	44%	50%	62%	54%	66%	50%	36%	70%	50%	55%
*SH3PXD2a*	0.69	0.72	0.63	1.64 ^#^	0.68	0.80	0.70	0.67	0.72	0.72	0.67	0.66
(TKS5)	±0.18	±0.17	±0.19	±0.71	±0.182	±0.11	±0.19	±0.17	±0.25	±0.15	±0.18	±0.18
NM_014631	70%	70%	70%	100%	72%	54%	68%	73%	64%	70%	75%	71%
*MMP14*	0.56	0.56	0.57	0.81	0.55	0.615	0.55	0.59	0.56	0.56	0.47	0.58
(MT1-MMP)	±0.22	±0.22	±0.24	±0.11	±0.22	±0.26	±0.23	±0.20	±0.23	±0.23	±0.19	±0.22
NM_004995	80%	86%	67%	100%	82%	62%	81%	77%	79%	91%	75%	74%

**Table 4:** Expression patterns of upregulated genes related to invadopodia formation in at least 60% of all OSCC samples compared to healthy mucosa (± = standard deviation; % = percent in OSCC samples, T = T status, G = grading, N = lymph nodes, UICC = UICC classification; * group G1 includes two samples, results are less robust.

Gene	All	T1 + T2	T3 + T4	G1 *	G2	G3	N0	N+	UICC1	UICC2	UICC3	UICC4
	n = 83	n = 56	n = 27	n = 2	n = 68	n = 13	n = 53	n = 30	n = 16	n = 23	n = 8	n = 36
*CTTN*	2.49	2.24	2.79	1.39	2.48	2.37	2.38	2.65	2.30	2.23	1.19	2.83
NM_005231	2.22	1.57	2.93		2.33	1.63	2.20	2.29	1.44	1.77	0.25	2.73
	69%	61%	85%	50%	71%	62%	74%	60%	79%	61%	50%	74%
*SRC*	1.63	1.54	1.80	0.86	1.70	1.35	1.57	1.73	1.42	1.49	2.19	1.68
NM_005417	0.73	0.73	0.72		0.80	0.22	0.56	0.96	0.49	0.48	1.78	0.64
	76%	75%	78%	50%	74%	92%	74%	80%	71%	74%	63%	82%
*EGFR*	5.28	5.58	4.70	2.10	5.33	5.51	4.45	6.80	3.21	5.95	2.38	6.26
NM_005228	7.86	8.81	5.72	0.72	8.00	8.03	5.27	11.11	2.44	7.50	1.17	9.84
	95%	93%	100%	100%	94%	100%	96%	93%	93%	96%	100%	95%
*PTK2*	1.71	1.70	1.72	1.60	1.68	1.80	1.70	1.72	1.39	1.94	1.26	1.76
NM_153831	0.72	0.78	0.60	0.50	0.76	0.48	0.75	0.66	0.33	1.05	0.38	0.61
	81%	79%	85%	100%	81%	81%	80%	83%	86%	72%	75%	87%
*MET*	3.28	3.55	2.79	0.91	2.88	5.82	3.39	3.11	4.72	2.92	2.27	3.20
NM_000245	3.78	4.54	1.44		1.59	8.94	4.52	2.00	8.70	1.23	1.05	1.84
	92%	90%	96%	50%	94%	87%	91%	93%	90%	93%	88%	93%
*STAT1*	7.68	6.45	10.20	2.04	7.87	7.59	7.14	8.66	5.85	6.65	7.07	9.15
NM_139266	7.45	6.75	8.25	1.54	7.35	8.46	7.84	6.80	5.30	7.39	6.51	8.29
Isoform beta	99%	98%	100%	100%	99%	100%	100%	97%	100%	100%	100%	97%
*STAT1*	4.49	3.96	5.56	1.835	4.56	4.27	4.19	5.03	3.16	4.10	4.73	5.22
NM_007315	3.32	2.98	3.73		3.26	3.78	3.34	3.25	2.45	2.88	3.86	3.66
Isoform alpha	94%	93%	96%	50%	94%	100%	94%	93%	100%	96%	88%	92%
*SYK*	2.39	2.21	2.69	1.655	2.05	2.40	2.30	2.53	2.37	2.03	2.16	2.63
NM_003177	0.89	0.80	0.97		1.07	0.92	0.94	0.82	0.86	0.79	0.49	0.95
	81%	75%	93%	50%	81%	85%	79%	83%	79%	78%	63%	87%
*WASL*	1.54	1.60	1.45	1.10	1.54	1.62	1.48	1.64	1.54	1.60	1.36	1.55
NM_003941	0.44	0.49	0.35		0.52	0.65	0.43	0.46	0.36	0.52	0.28	0.47
	64%	57%	78%	100%	63%	62%	62%	67%	57%	57%	75%	68%
*GRB2*	1.67	1.60	1.81	1.79	1.68	1.63	1.66	1.71	1.61	1.57	1.36	1.79
NM_002086	0.54	0.50	0.60	0.24	0.57	0.42	0.59	0.46	0.40	0.57	0.25	0.57
	85%	83%	90%	100%	85%	87%	86%	83%	93%	80%	67%	89%
*MAPRE1*	2.75	2.62	3.01	2.16	2.78	2.64	2.78	2.70	2.60	2.61	2.20	3.02
NM_012325	1.31	1.17	1.55		1.39	0.89	1.31	1.34	1.40	1.00	1.03	1.48
	98%	97%	100%	50%	99%	96%	98%	98%	96%	100%	100%	97%
*PLAUR*	4.12	4.10	4.15	5.11	3.94	4.02	3.95	4.42	3.04	4.68	3.30	4.29
NM	3.60	4.04	2.61	5.78	3.90	2.21	4.05	2.70	1.00	5.75	1.58	2.61
_00100537	93%	91%	96%	100%	93%	92%	92%	93%	79%	96%	100%	95%
*IQGAP1*	1.40	1.30	1.64	1.11	1.43	1.33	1.39	1.42	1.19	1.32	1.10	1.57
NM_003870	0.61	0.35	0.98	0.22	0.67	0.37	0.69	0.40	0.22	0.36	0.13	0.81
	61%	64%	56%	100%	57%	77%	66%	53%	57%	70%	38%	63%
*FLNA*	2.08	1.92	2.41	0.885	1.61	2.75	1.93	2.36	2.00	1.99	1.44	2.23
NM_001456	1.62	1.08	2.37		1.13	3.21	1.08	2.33	1.45	1.22	0.25	1.96
	65%	64%	67%	50%	63%	77%	66%	63%	57%	70%	33%	71%
*WIPF1*	2.30	2.15	2.61	2.46	2.28	2.38	2.12	2.60	2.74	1.75	2.05	2.47
NM_003387	1.84	1.96	1.58	1.37	1.97	1.02	2.00	1.55	3.57	0.58	0.91	1.45
	80%	79%	81%	100%	82%	69%	77%	85%	86%	72%	81%	83%
*WASL*	1.54	1.60	1.45	1.10	1.54	1.62	1.48	1.64	1.54	1.60	1.36	1.55
(N-WASP)	0.44	0.49	0.35	0.08	0.41	0.65	0.43	0.46	0.36	0.52	0.28	0.47
NM_003941	64%	57%	78%	100%	63%	62%	62%	67%	57%	57%	75%	68%
*SH3PXD2B*	2.13	2.16	2.08	1.82	2.14	2.15	2.06	2.27	1.76	2.27	2.39	2.14
(TKS4) NM_	0.95	1.03	0.73	0.63	1.00	0.70	0.98	0.88	0.73	1.25	0.73	0.80
001017995	95%	96%	94%	100%	95%	96%	95%	95%	96%	98%	88%	95%
*FSCN1*	13.94	12.44	16.93	2.88	13.71	15.81	13.41	14.88	11.58	12.55	9.10	16.82
NM_003088	8.64	7.92	9.37		8.12	11.63	8.63	8.74	7.64	8.04	6.32	9.11
	98%	96%	100%	50%	99%	100%	98%	97%	100%	100%	100%	95%
*AFAP1*	1.51	1.51	1.53	1.49	1.47	1.68	1.42	1.69	1.38	1.42	1.45	1.65
NM_021638	0.52	0.46	0.68	0.10	0.46	0.71	0.44	0.61	0.55	0.39	0.29	0.61
	60%	66%	48%	100%	54%	85%	60%	60%	64%	65%	63%	55%
*ARPC2*	1.29	1.29	1.29	0.8	1.28	1.35	1.15	1.31	1.36	1.25	1.13	1.32
NM_152862	0.29	0.31	0.28		0.31	0.39	0.35	0.35	0.36	0.21	0.25	0.31
	69%	61%	85%	50%	69%	69%	68%	70%	57%	57%	88%	76%

## Data Availability

The datasets analyzed during the current study are available from the corresponding author on reasonable request.
